# Shared diagnostic genes and potential mechanism between allergic rhinitis and atopic dermatitis revealed by integrated transcriptomic analysis and machine learning

**DOI:** 10.3389/falgy.2025.1686370

**Published:** 2025-11-21

**Authors:** Xiaojing Zhang, Meng Sun, Liang Xu, Junjie Bi, Xiangjing Chen, Lei Yao, Yuan Jia, Ying Zhang, Wei Meng

**Affiliations:** 1 Department of Otolaryngology of Traditional Chinese Medicine, Shandong University of Traditional Chinese Medicine, Jinan, Shandong, China; 2 Department of Otolaryngology, Affiliated Hospital of Shandong University of Traditional Chinese Medicine, Jinan, Shandong, China; 3 Department of Otolaryngology Head and Neck Surgery, Central Hospital Affiliated to Shandong First Medical University, Jinan, Shandong, China; 4 Department of Healthcare, Affiliated Hospital of Shandong University of Traditional Chinese Medicine, Jinan, Shandong, China; 5 The First Clinical Medical College of Shandong University of Traditional Chinese Medicine, Jinan, Shandong, China

**Keywords:** allergic rhinitis, atopic dermatitis, biomarkers, miRNA, immune

## Abstract

**Introduction:**

Allergic rhinitis (AR) and atopic dermatitis (AD) frequently co-occur, yet their shared molecular mechanisms are poorly understood. We used an integrative bioinformatics approach to identify common diagnostic biomarkers and mechanistic links between them.

**Methods:**

Transcriptomic datasets from AR and AD patients were analyzed to identify overlapping differentially expressed genes (DEGs). Hub genes were subsequently identified using protein-protein interaction (PPI) networks and random forest modeling, followed by functional enrichment and immune infiltration analyses.

**Results:**

Our analysis identified 36 overlapping DEGs between AR and AD. From these, five hub genes—CD274, CYP2E1, FOLH1, SERPINB4, and SPRR1B—were revealed, all of which demonstrated strong diagnostic value in both diseases. Functional analysis indicated their involvement in epithelial barrier regulation, immune cell signaling, and oxidative stress pathways. Immune infiltration profiling showed a significant association between these genes and dendritic cells, T cells, and natural killer cells in both AR and AD cohorts.

**Conclusion:**

AR and AD share a common molecular landscape, and the five hub genes identified here represent robust biomarkers for diagnosis and potential therapeutic targets for these interconnected diseases.

## 1 Introduction

Allergic rhinitis (AR) and atopic dermatitis (AD) are among the most prevalent allergic disorders worldwide, and their incidence has risen markedly in recent decades (1). AR affects up to 10%–40% of the population, amounting to over 400 million sufferers globally (2). It is an IgE-mediated inflammatory condition of the nasal mucosa, clinically characterized by paroxysms of sneezing, rhinorrhea, nasal itching, and congestion (3). Despite being non-life-threatening, AR is a global health problem that imposes a substantial burden and disability, significantly impairing sleep, cognitive function, and daily productivity, particularly among working-age adults and school-aged children (4, 5). AD is a chronic relapsing inflammatory skin disease marked by eczematous lesions, intense pruritus, and xerosis. This dermatosis affects up to 20% of children and approximately 10% of adults, ranking as the leading cause of skin disease burden globally in terms of disability-adjusted life years (6, 7). The intense and persistent itch of AD severely disrupts sleep and psychosocial well-being, contributing to high rates of anxiety, depression, and reduced school and work performance (8). Both AR and AD pose substantial clinical and socioeconomic burdens, impairing quality of life and productivity.

Epidemiological studies have revealed a frequent co-occurrence of AR and AD within the same individuals, suggesting shared pathogenic mechanisms and genetic susceptibilities (9). Central to the pathophysiology of both diseases is the dysregulation of type 2 helper T (Th2) cell-mediated immune responses (10). This immune skewing leads to elevated production of cytokines such as IL-4, IL-5, and IL-13, promoting immunoglobulin E (IgE) synthesis and eosinophilic inflammation (11). These cytokines not only exacerbate inflammatory responses but also impair epithelial barrier functions in the skin and nasal mucosa, facilitating allergen penetration and perpetuating a cycle of inflammation and barrier disruption (12).

The concept of the “atopic march” describes the typical progression of allergic diseases, in which AD in early childhood often precedes the later development of AR and other allergic conditions. This sequential manifestation underscores the epidemiological and immunological interconnection of these disorders and highlights the importance of elucidating their shared molecular mechanisms (1). Despite advances in our understanding of allergic inflammation, the precise molecular pathways linking AD and AR remain insufficiently defined.

In this study, we aim to employ integrative bioinformatics approaches to identify common differentially expressed genes and signaling pathways involved in both AR and AD. By analyzing transcriptomic datasets from patients with these conditions, we seek to uncover shared molecular signatures that could elucidate the overlapping pathophysiology of AR and AD. Identifying such biomarkers and pathways may provide insights into potential therapeutic targets and inform strategies for early intervention and prevention in individuals predisposed to the atopic march.

## 2 Methods

### 2.1 Public data source acquisition and pre-processing

The gene expression matrices and corresponding clinical data for AR and AD were retrieved from the Gene Expression Omnibus (https://www.ncbi.nlm.nih.gov/geo/). For AD, transcriptome microarray data of 27 AD-affected skin samples and 38 healthy control samples were obtained from GSE121212 as the primary analysis dataset. In addition, 13 AD samples and 8 healthy controls from GSE32924 were used as an independent validation cohort. For AR, GSE19187 contained transcriptome microarray data of 14 AR nasal epithelial samples and 11 healthy controls, and GSE43523 included 7 AR samples and 5 controls as a validation dataset (Supplementary Table S1). To eliminate batch effects, the “NormalizeBetweenArrays” function of the “limma” package in the R programming language was employed (13).

### 2.2 Differential expression gene analysis

Differential expression was assessed using the limma package on log^2^-transformed expression values, which fulfill the model's approximate normality assumption. For discovery, we defined DEGs as genes with *P* < 0.05 and |log2FC| >0.5. This nominal *p*-value criterion was chosen to avoid over-stringent filtering in heterogeneous public cohorts. Gene set enrichment used standard multiple-testing control at the pathway level. Volcano plots were generated with genekitr (14).

### 2.3 Pathway enrichment analysis

To explore the biological functions and signaling pathways associated with DEGs, we conducted Gene Ontology (GO) and Kyoto Encyclopedia of Genes and Genomes (KEGG) pathway enrichment analyses using the “clusterProfiler” R package (15). Results with significantly enriched terms (*P*.adjust <0.05) were visualized using the “genekitr” R package. Additionally, gene set enrichment analysis (GSEA) was performed to identify pathway enrichment in patients with varying levels of gene expression. Hallmark pathways were utilized for GSEA, which was also conducted using the “genekitr” R package. Enrichment results with *P*.adjust <0.05 were considered statistically significant.

### 2.4 Protein–protein interaction network analysis

Proteins interact to form functional networks, where a higher number of interactions generally indicates greater biological centrality. The 36 shared DEGs between AR and AD were queried in STRING v12.0 (https://string-db.org) to construct a protein–protein interaction/functional association network (16). Unless otherwise specified, default parameters were used: all evidence channels enabled (experiments, curated databases, co-expression, neighborhood, gene fusion, co-occurrence, and text mining), minimum required interaction score = 0.40 (medium confidence), network type = evidence, and no additional interactors. In the resulting network, the degree score was defined as the number of direct connections (edges) a protein has to other proteins. The average node degree was calculated by STRING, and proteins with a degree greater than or equal to the average were designated as core proteins. Twelve core proteins were identified and taken forward for downstream analyses.

### 2.5 Identification of core diagnostic genes using machine learning

To identify disease-specific feature genes using machine learning, we employed the “RandomForest” R package to classify important genes through the random forest (RF) algorithm. RF analysis, based on a decision tree approach, identified the most significant variables. A RF model with 500 trees was constructed for the corresponding cohort, and the optimal number of trees was determined by minimizing the cross-validation error. Genes were ranked by importance, and the top eight most important genes were selected as feature genes for AR or AD. Finally, we identified the intersection of disease-specific feature genes determined by RF for each disease.

### 2.6 Immune infiltration analysis

The “IOBR” R package was used to estimate immune cells in the AR and AD cohorts (17). Specifically, the MCPcounter algorithm was employed to quantify the relative immune cell fractions in individual samples. No specific parameters were manually set. Student's *t*-test was used for statistical analysis of immune cell differences between groups, and Pearson correlation was applied to calculate correlations.

### 2.7 Construction of drug–gene target and mRNA–miRNA network

For the analysis of the drug–target network, the DGIdb database was employed to identify the targets of relevant genes (18). The DGIdb database, a comprehensive resource for drug–gene interactions, aggregates data from multiple sources. It not only offers information on known interactions between genes and drugs but also provides insights into potential associations. This includes data from preclinical research, clinical trials, and pharmacological databases. After retrieving and organizing the information on relevant targets and drugs from the DGIdb database, Cytoscape was utilized to construct the drug–target network. Similarly, the mRNA–miRNA regulatory network was constructed based on the miRDB database (https://mirdb.org) and was further visualized using Cytoscape software.

### 2.8 Sample collection, RNA extraction, and qPCR

Peripheral blood samples were collected in EDTA-coated tubes from patients with concomitant AR and AD (*n* = 5) and healthy donors (*n* = 5) (Supplementary Table S1). Participants were 30–42 years of age with a male–female ratio of 1:1. All samples were processed within 2 h of collection to ensure RNA integrity. Total RNA was extracted from the blood samples using the MolPure® Blood RNA Kit (Yeasen, China) following the manufacturer's protocols. The extracted RNA was then used for first-strand cDNA synthesis with the Transcriptor High Fidelity cDNA Synthesis Kit (Roche, Switzerland). For PCR analysis, Green Taq Mix (TAKARA, China) was utilized in accordance with the manufacturer's instructions. The primers used in this study were as follows: FOLH1 forward: 5′-TTGACAAAAGCAAGCATGTCA-3′, reverse: 5′-TTCCTGGGAATGACTCCCCT-3′; SERPINB4 forward: 5′-CGATGGTCTGCAGAAGCTTGA-3′, reverse: 5′-TTCCTGGGAATGACTCCCCT-3′; SPRR1B forward: 5′-ATGCATCCCCAAAACCAAGG-3′, reverse: 5′-GCTGGTGCTGGAGTGACTAT-3′; CD274 forward: 5′-CATTTGCTGAACGCCCCATA-3′, reverse: 5′-TCCAGATGACTTCGGCCTTG-3′; CYP2E1 forward: 5′-CACAGGGACAGGGGAATCAT-3′, reverse: 5′-ATAGTTCCGGAGGGTGGTCA-3′; GAPDH forward: 5′-AGGGCTGCTTTTAACTCTGGT-3′, reverse: 5′-CCCCACTTGATTTTGGAGGGA-3′. For qRT-PCR analysis, the SYBR® Green Premix Kit (TAKARA, China) was used according to the manufacturer's instructions.

### 2.9 Statistical analysis

Data processing, analysis, and visualization were carried out using R packages (version 4.30; https://www.bioconductor.org/) or GraphPad Prism software (version 8.0.1; https://www.graphpad.com/). To compare differences between two groups, we employed Student's *t*-test. Correlation analysis between variables was performed using Pearson test. A significance level of *P* < 0.05 was considered statistically significant. The results are expressed as the mean ± standard error of the mean.

## 3 Results

### 3.1 Identification of DEGs in allergic rhinitis and atopic dermatitis

**Figure 1:**
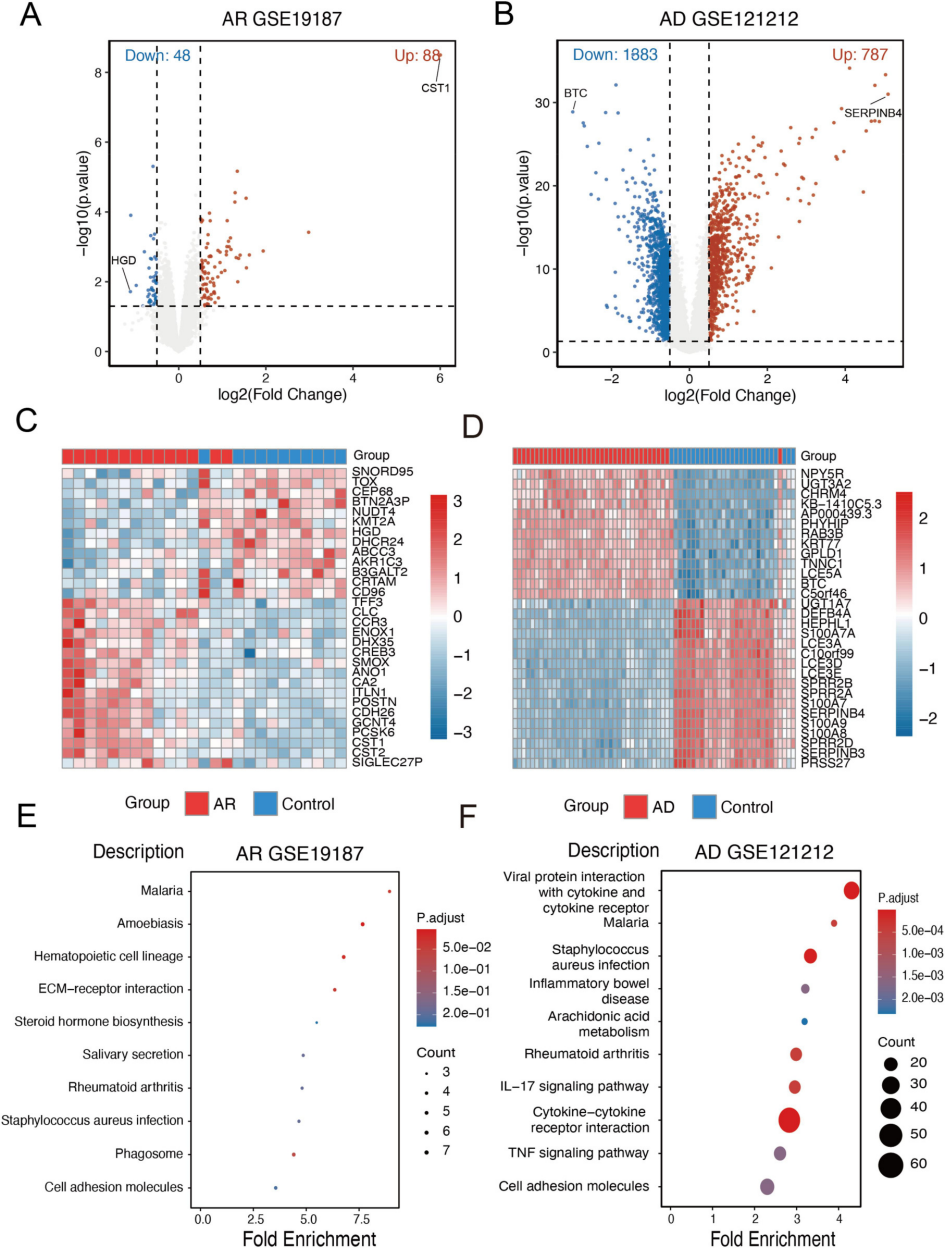
Identification of DEGs in allergic rhinitis and atopic dermatitis. **(A)** Volcano plot of DEGs in the AR group compared with the control. **(B)** Volcano plot of DEGs in the AD group compared with the control. **(C)** Heatmap depicting hierarchical clustering of the top significantly DEGs between the AR and control groups. **(D)** Heatmap depicting hierarchical clustering of the top significantly DEGs between the AD and control groups. **(E)** KEGG pathway enrichment analysis showing significantly enriched pathways based on DEGs of AR. **(F)** KEGG pathway enrichment analysis showing significantly enriched pathways based on DEGs of AD.

To investigate the potential molecular overlap between AR and AD, we first identified DEGs in each disease group. Using linear modeling with the “limma” R package, a total of 136 DEGs were identified in the AR dataset (GSE19187), including 88 upregulated and 48 downregulated genes (Figure 1A). In the AD dataset (GSE121212), a total of 2,170 DEGs were detected, with 787 genes upregulated and 1,383 downregulated compared with healthy controls (Figure 1B). To further visualize the distribution and expression patterns of these DEGs, heatmaps were generated to display the top 30 most significantly dysregulated genes in both the AR and AD groups (Figures 1C,D). Subsequent KEGG pathway enrichment analysis was conducted to explore the biological functions of these DEGs. In the AR group, the top enriched pathways included extracellular matrix (ECM)–receptor interaction, steroid hormone biosynthesis, hematopoietic cell lineage, and rheumatoid arthritis, implicating pathways related to ECM remodeling and immune activation (Figure 1E). In contrast, the DEGs from the AD group were significantly enriched in immune-related signaling pathways, such as the IL-17 signaling pathway, cytokine–cytokine receptor interaction, tumor necrosis factor (TNF) signaling pathway, and cell adhesion molecules (Figure 1F). These immune and inflammatory pathways are consistent with known mechanisms underlying allergic and atopic conditions.

### 3.2 Identification of core interacting proteins shared by allergic rhinitis and atopic dermatitis

**Figure 2:**
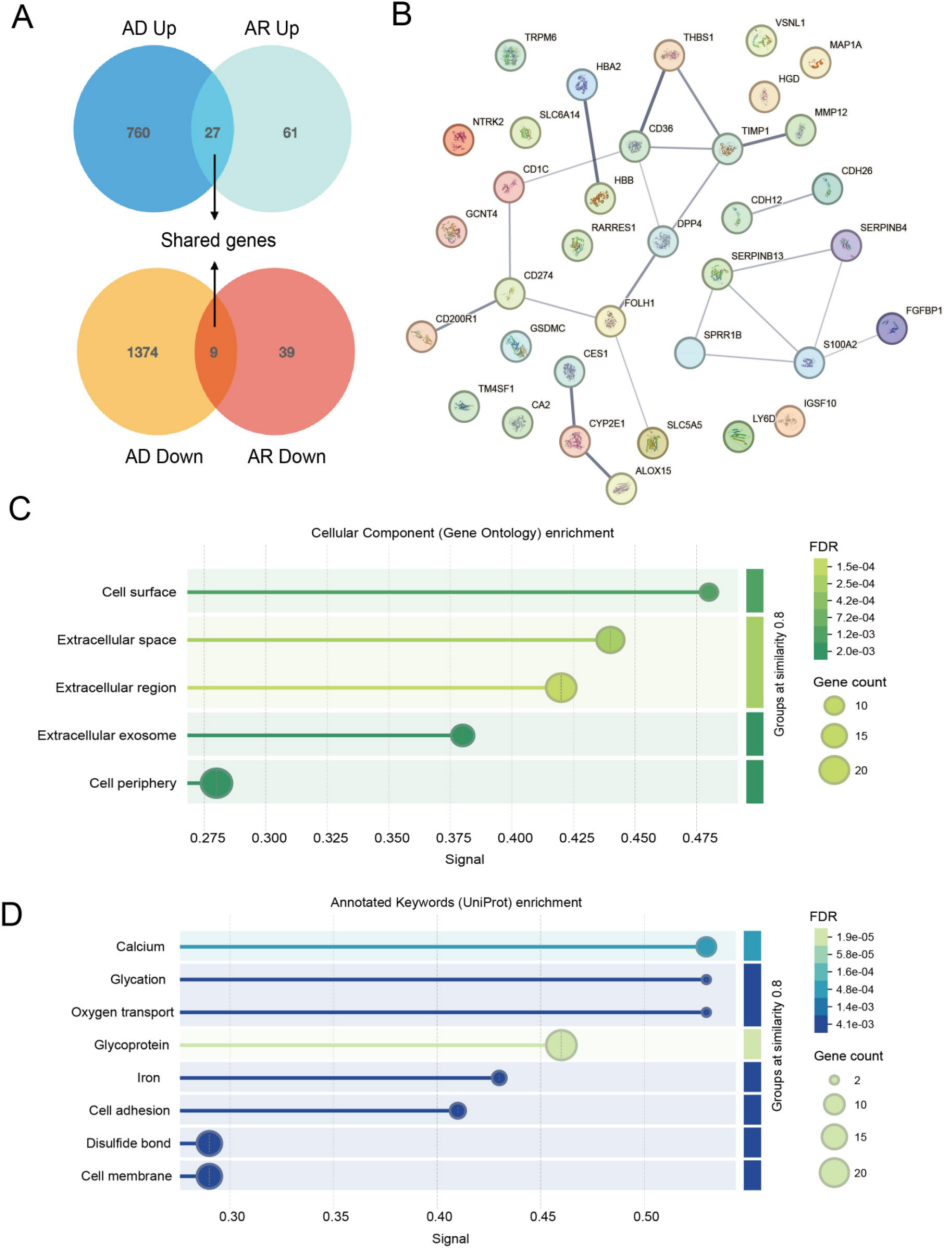
Identification of core interacting proteins shared by allergic rhinitis and atopic dermatitis. **(A)** Determination of shared DEGs among allergic rhinitis and atopic dermatitis. **(B)** PPI network of overlap genes. **(C)** Dot plot of GO–CC pathway enrichment for core interaction proteins. **(D)** Dot plot of UniProt pathway enrichment for core interaction proteins.

To investigate common molecular mechanisms underlying AR and AD, we identified 36 shared DEGs that were either co-upregulated or co-downregulated across both conditions (Figure 2A). These overlapping DEGs likely represent key mediators involved in the shared pathophysiology of the two allergic diseases. To further explore the functional relationships among these genes, a PPI network was constructed using the STRING database. The resulting network revealed a set of interconnected proteins, among which 12 core hub proteins were identified based on a node degree greater than or equal to the average connectivity, including CD36, S100A2, TIMP1, CD274, DPP4, FOLH1, SERPINB13, CD1C, CYP2E1, SERPINB4, SPRR1B, and THBS1 (Figure 2B). These core proteins may represent central regulators within the shared molecular network of AR and AD. GO enrichment analysis of the cellular component (CC) category showed that these core proteins were significantly enriched in components such as the cell surface, extracellular space, and extracellular region (Figure 2C), indicating their involvement in intercellular signaling and immune communication. Additionally, UniProt pathway enrichment analysis highlighted functional enrichment in calcium ion transport, glycation-related processes, and oxygen transport pathways, suggesting that these proteins may participate in diverse cellular physiological functions relevant to both diseases (Figure 2D). Collectively, these findings underscore the functional importance of the shared DEGs and their encoded proteins in mediating extracellular interactions, metabolic responses, and immune signaling in the context of AR and AD comorbidity.

### 3.3 Identification of potential shared diagnostic genes based on machine learning

**Figure 3:**
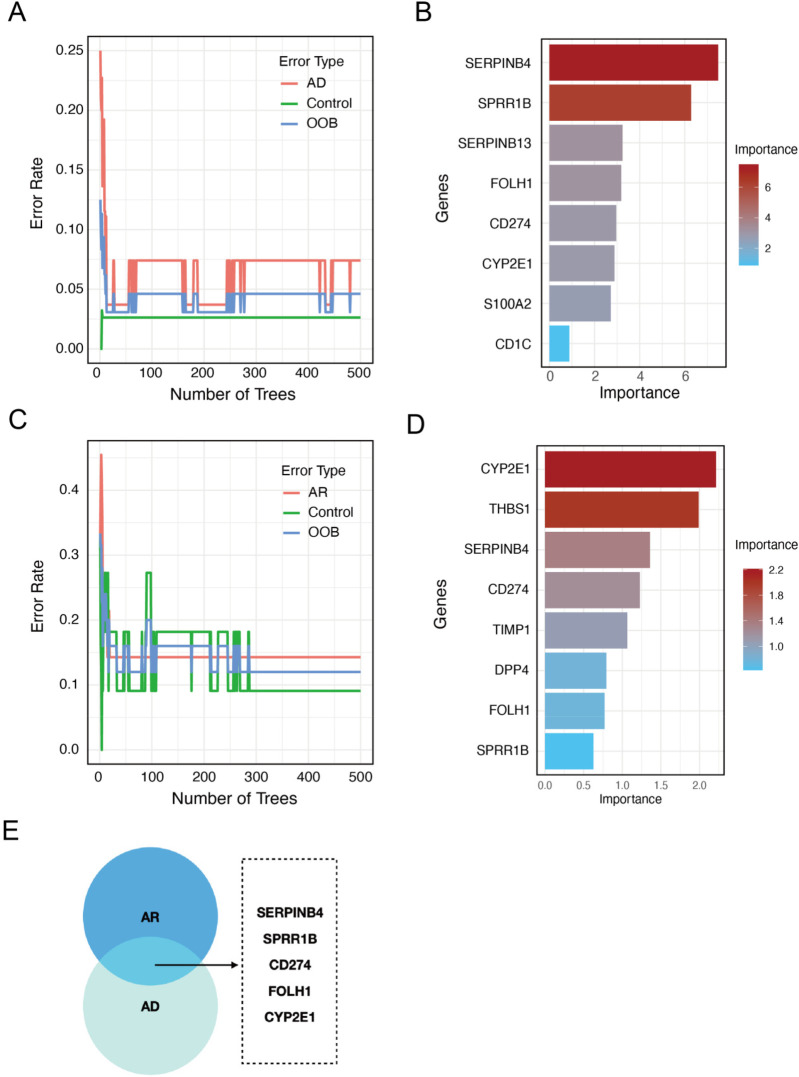
Identification of potential shared diagnostic genes based on machine learning. **(A)** Curve of RF error rate in the AD group. The line plot displays the error rate of the RF model applied to the AD group. The *y*-axis represents the error rate, while the *x*-axis indicates the number of decision trees used in the model. **(B)** The top eight most important genes identified by RF in the AD group. **(C)** Line plot of RF error rate in the AR group. The line plot displays the error rate of the RF model applied to the AR group. The *y*-axis represents the error rate, while the *x*-axis indicates the number of decision trees used in the model. **(D)** Bar plot of the top eight genes ranked by importance in the AR group. **(E)** Venn diagram showing the overlap of RF-identified genes in both AD and AR.

For further selection of the most promising candidate diagnostic gene targets with significant potential for classifying the disease and control groups, we applied RF algorithms based on the 12 core interaction proteins. In the AD group, the 12 core interaction proteins were input into the RF classifier, where the top eight genes were ranked according to their importance (Figures 3A,B). The corresponding out-of-bag error is reported in Supplementary Table S2. Similarly, in the AR group, the RF machine learning screening process identified the top eight potential biomarkers (Figures 3C,D). By overlapping the top eight important genes identified in the AD and AR groups, we identified SERPINB4, SPRR1B, CD274, FOLH1, and CYP2E1 as conserved disease-shared genes for potential core diagnostic genes (Figure 3E).

### 3.4 Expression validation of core diagnostic genes

**Figure 4:**
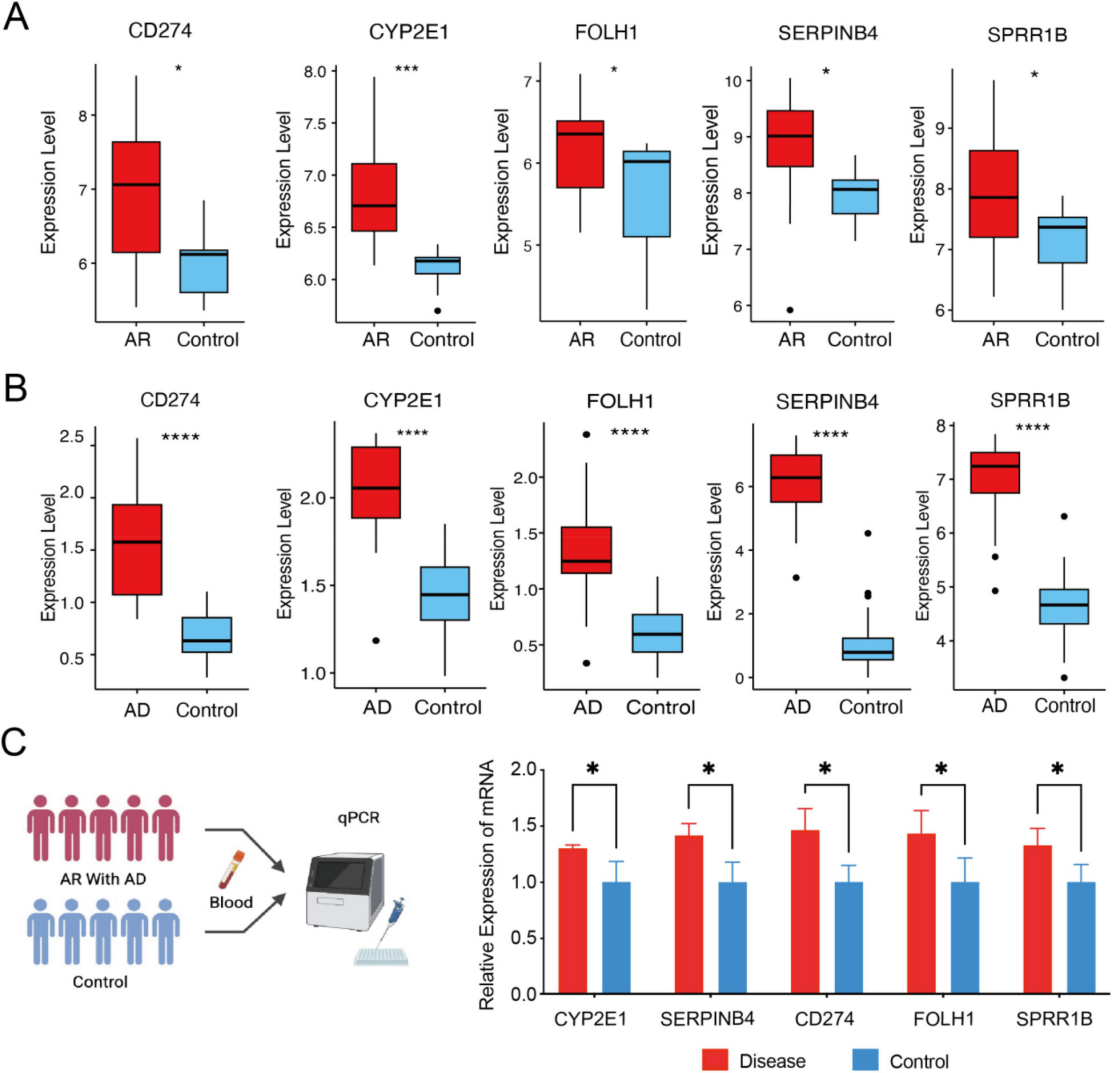
Expression validation of core diagnostic genes. **(A)** Box plot of core diagnostic gene expression in the AR group and control. **(B)** Box plot of core diagnostic gene expression in the AD and control groups. **(C)** qPCR validation of the diagnostic efficacy of core diagnostic genes in clinical patients (*n* = 5 vs. 5). Statistical significance was assessed using Student's *t*-test. (*P < 0.05, **P < 0.01, ***P < 0.001).

We further validated the expression patterns of the five core diagnostic genes (SERPINB4, SPRR1B, CD274, FOLH1, and CYP2E1) using the corresponding datasets for AR (GSE19187) and AD (GSE121212). Compared with healthy control samples, all five genes were significantly upregulated in both the AR cohort (Figure 4A) and the AD cohort (Figure 4B) (*P* < 0.05), which is consistent with our previous findings. Additionally, in our clinical validation cohort, peripheral blood from patients with concomitant AR and AD (*n* = 5) and from healthy donors (*n* = 5) was analyzed. The expression levels of CYP2E1, SERPINB4, CD274, FOLH1, and SPRR1B were significantly higher in the comorbid group than in healthy controls (Figure 4C). These results support that the five core genes can serve as shared diagnostic markers for AD and AR.

### 3.5 Diagnostic value and validation of core diagnostic genes

**Figure 5:**
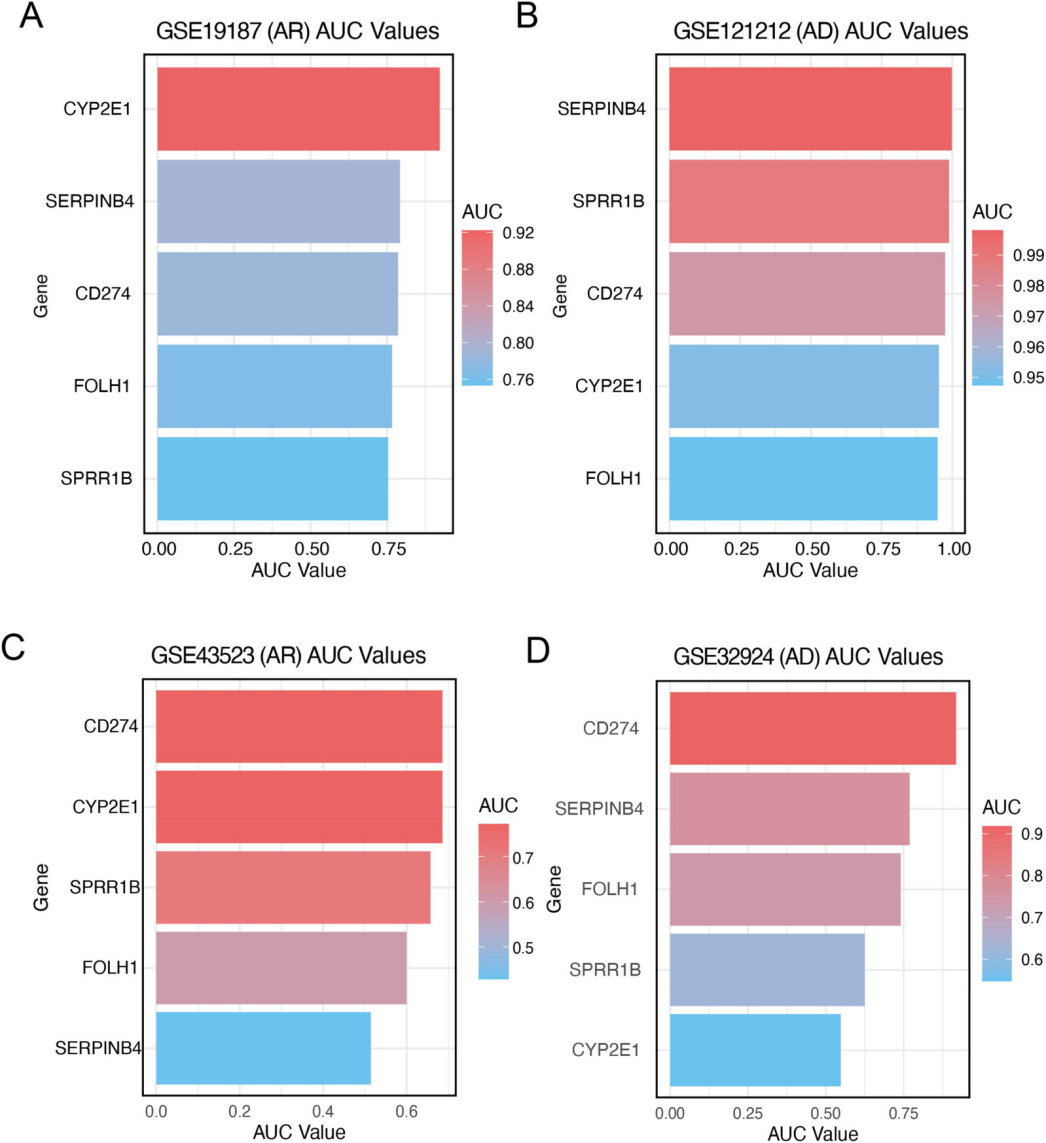
Diagnostic value and validation of core diagnostic genes. **(A)** Bar plot showing the diagnostic accuracy of each candidate gene measured by AUC values in GSE19187. **(B)** Bar plot showing the diagnostic accuracy of each candidate gene measured by AUC values in GSE121212. **(C)** Bar plot showing the diagnostic accuracy of each candidate gene measured by AUC values in GSE43523. **(D)** Bar plot showing the diagnostic accuracy of each candidate gene measured by AUC values in GSE32924.

To quantify the diagnostic performance of the core genes shared by AR and AD, we conducted ROC analyses across independent transcriptomic cohorts; the ROC curves with their corresponding AUC values are provided in Supplementary Figure S1. In the AR discovery cohort (GSE19187), the five hub genes—CYP2E1, SERPINB4, CD274, FOLH1, and SPRR1B—showed moderate-to-high discriminative ability (AUC 0.75–0.85), with CYP2E1 performing best (Figure 5A). In the AD discovery cohort (GSE121212), all five genes achieved excellent performance (AUC >0.95), and SERPINB4, SPRR1B, and CD274 approached near-perfect classification (AUC >0.98) (Figure 5B). External validation corroborated these findings: in the AR validation cohort (GSE43523), CD274, CYP2E1, and SPRR1B maintained strong performance, whereas SERPINB4 and FOLH1 showed moderate discrimination (Figure 5C). In the AD validation cohort (GSE32924), CD274 ranked highest (AUC ∼0.90), followed by SERPINB4, FOLH1, and SPRR1B, confirming consistent diagnostic utility across datasets (Figure 5D).

### 3.6 Construction nomograms for allergic rhinitis and atopic dermatitis diagnosis

**Figure 6:**
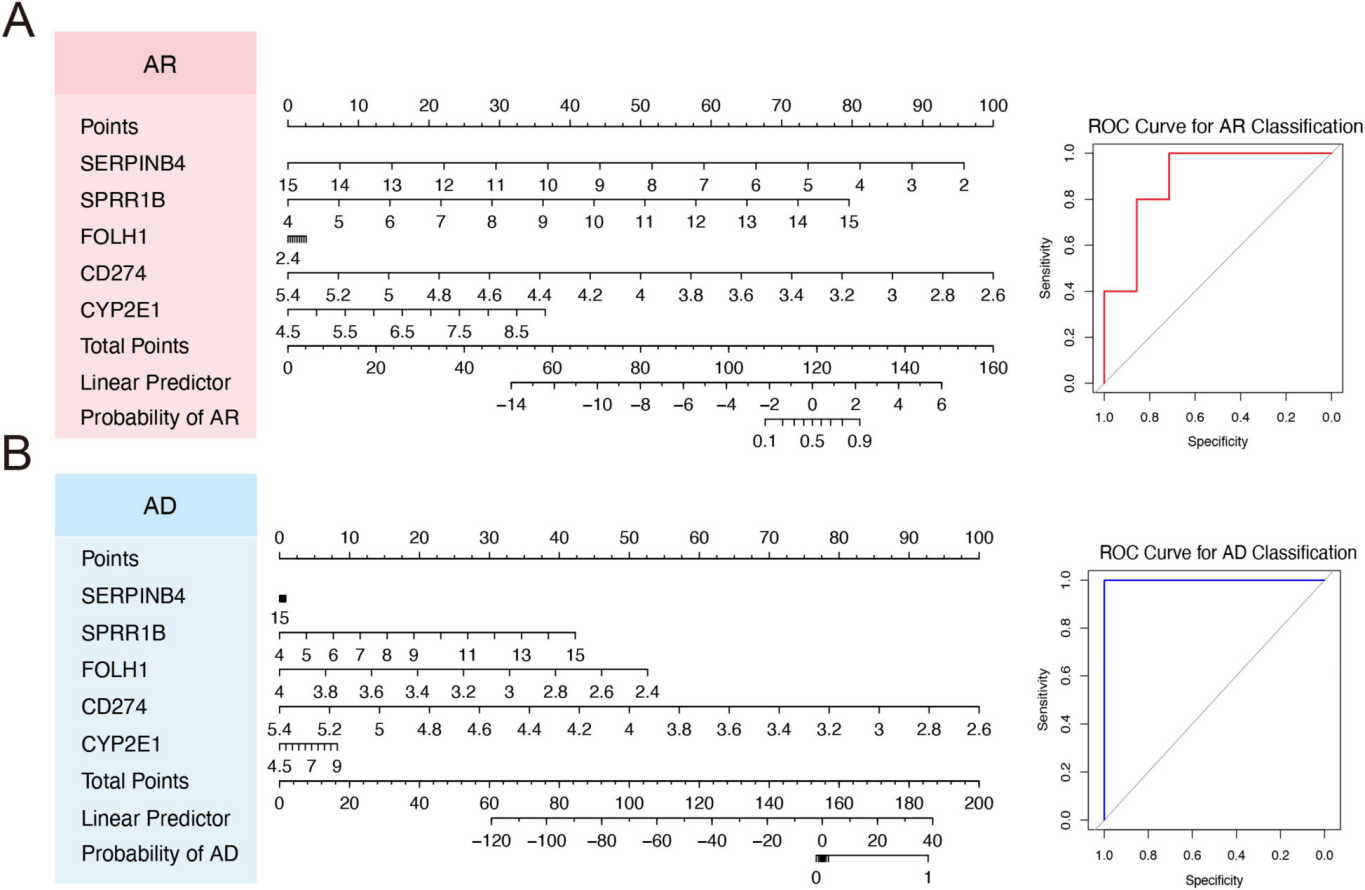
Construction nomograms for allergic rhinitis and atopic dermatitis diagnosis. **(A)** Nomogram model for AR based on five diagnostic hub genes (SERPINB4, SPRR1B, FOLH1, CD274, and CYP2E1). Each gene corresponds to a specific point value on the topmost scale. The ROC on the right demonstrates the model's diagnostic performance in AR classification. **(B)** Nomogram model for AD. The ROC curve on the right shows near-perfect classification accuracy, supporting the model's strong diagnostic utility in the AD dataset.

To further evaluate the clinical applicability of the identified hub biomarkers, diagnostic nomogram models were constructed for both AR and AD based on the five shared genes: SERPINB4, SPRR1B, FOLH1, CD274, and CYP2E1. For the AR cohort, each gene was assigned a weighted score proportional to its contribution to disease classification. The sum of the individual gene scores produced a total point value, which was then translated into a linear predictor and corresponding disease probability (Figure 6A). The ROC analysis demonstrated strong diagnostic accuracy for the nomogram model, indicating effective discrimination between AR patients and controls (Figure 6A). Similarly, a diagnostic nomogram for AD was developed using the same five genes. The scoring system and probabilistic output followed an analogous structure (Figure 6B). Notably, the AD-specific model achieved near-perfect performance, as evidenced by the ROC curve, which yielded an AUC approaching “1.0” (Figure 6B). These results underscore the potential utility of these shared hub genes in constructing robust, interpretable models for the diagnosis of both AR and AD. The consistent predictive power across conditions supports their role as valuable molecular markers in the clinical stratification of allergic diseases.

### 3.7 Pathway enrichment associated with diagnostic hub biomarkers

**Figure 7:**
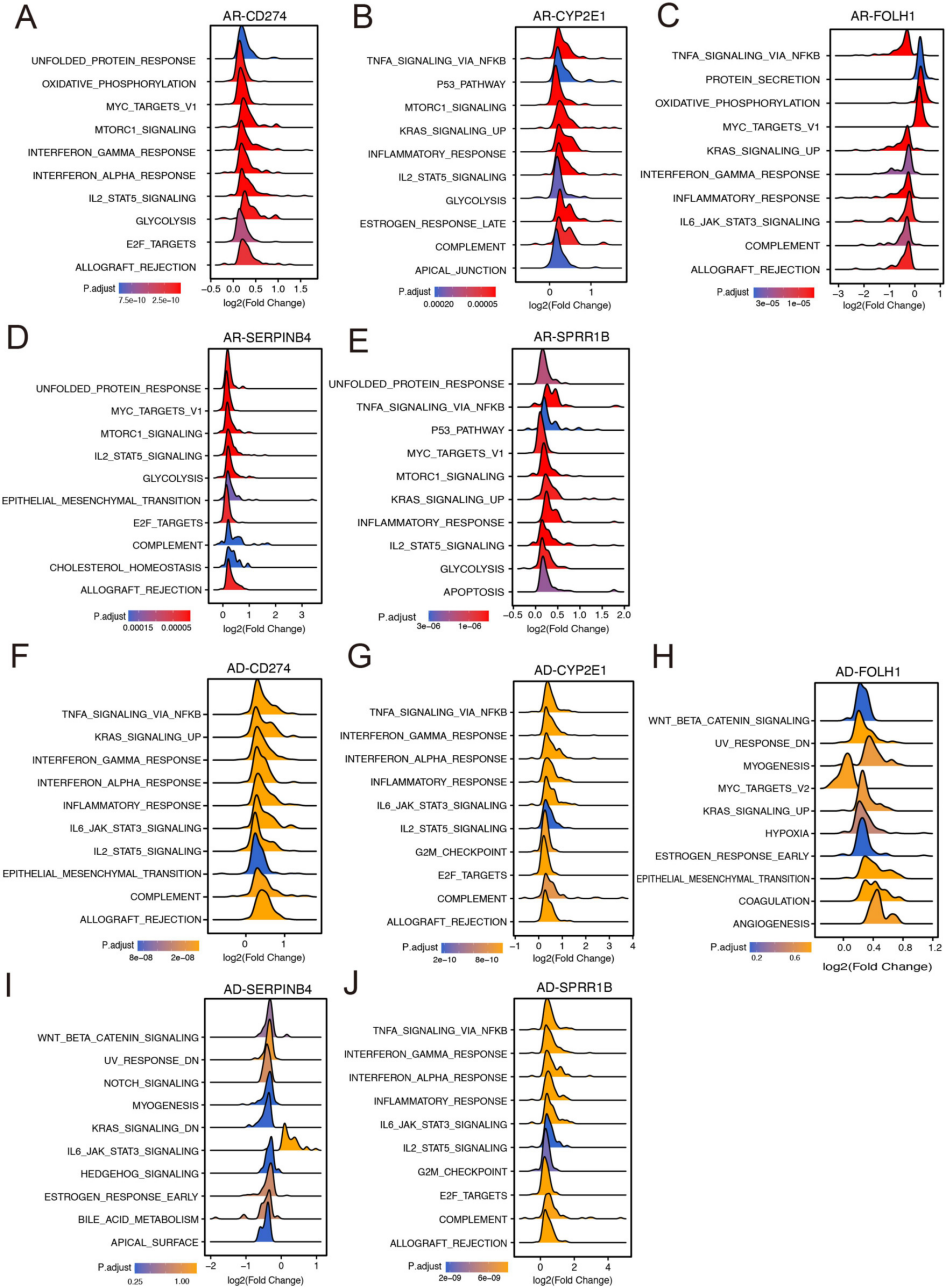
Pathway enrichment associated with diagnostic hub biomarkers. **(A–E)** GSEA ridge plots illustrating enriched Hallmark pathways associated with diagnostic hub genes in AR. **(F–J)** GSEA ridge plots illustrating enriched Hallmark pathways associated with diagnostic hub genes in AD.

To explore the functional implications of the identified diagnostic hub genes in AR and AD, we conducted GSEA using hallmark pathway gene sets. The analysis revealed that these hub genes are strongly associated with immune and inflammatory signaling pathways across both conditions. In the AR dataset, CD274, CYP2E1, and SPRR1B were notably enriched in pathways related to TNF-α signaling via NF-κB, IL-2/STAT5 signaling, and inflammatory response, highlighting their involvement in proinflammatory immune regulation (Figures 7A–E). Additionally, pathways such as unfolded protein response, mTORC1 signaling, and oxidative phosphorylation were recurrently enriched among AR hub genes, suggesting a role for cellular stress responses and metabolic dysregulation in AR pathogenesis. In the AD cohort, similar pathway enrichment patterns were observed. CD274 and CYP2E1 were again associated with NF-κB, interferon gamma, and IL6/JAK/STAT3 signaling, indicating conserved immune-inflammatory axes between AR and AD (Figures 7F,G). Moreover, genes such as SERPINB4 and FOLH1 in AD showed enrichment in Wnt/β-catenin signaling, epithelial–mesenchymal transition, and angiogenesis, reflecting the broader involvement of tissue remodeling and barrier dysfunction in AD pathophysiology (Figures 7H,I).

### 3.8 Immune infiltration analysis reveals the cross talk between the core diagnostic genes and atopic dermatitis as well as allergic rhinitis

To further elucidate the immunological relevance of the identified diagnostic biomarkers, we evaluated the correlation between gene expression and immune cell infiltration using the MCPcounter algorithm in both AR and AD cohorts. Most hub genes displayed significant associations with key immune cell populations, particularly myeloid dendritic cells, T cells, and natural killer cells.

**Figure 8:**
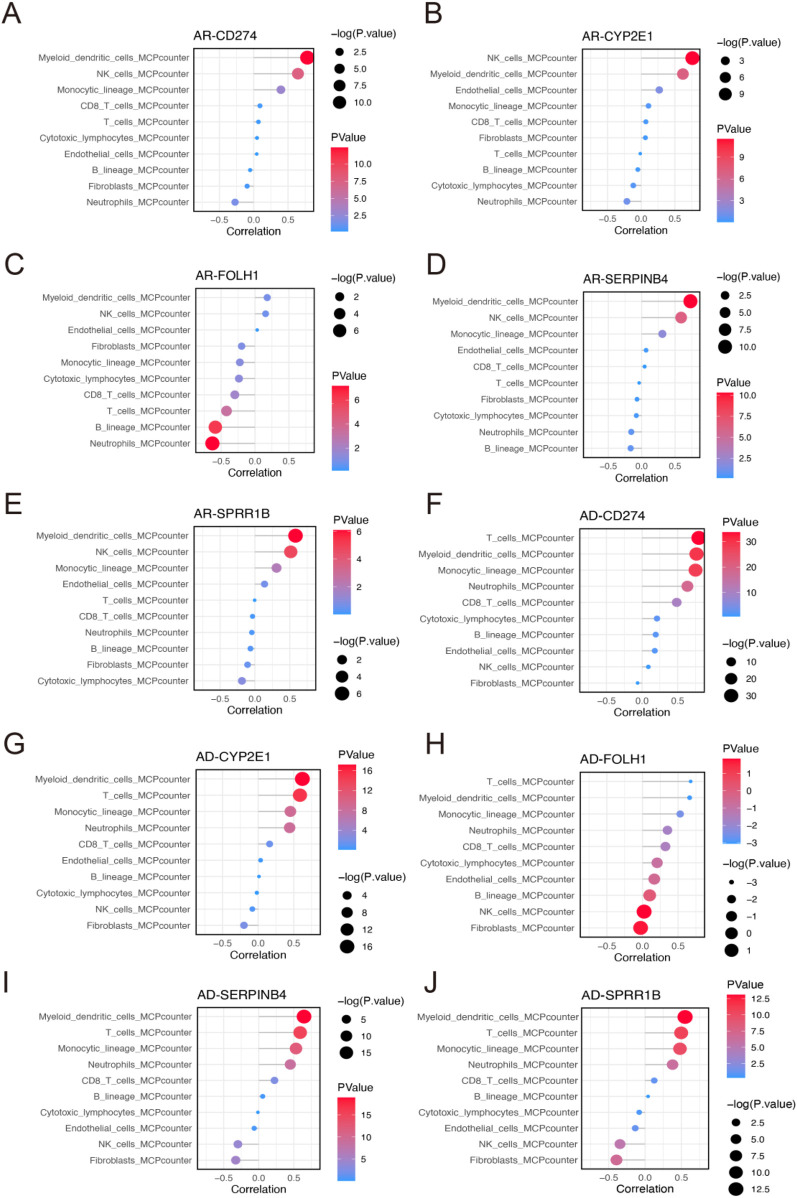
Immune infiltration analysis reveals the crosstalk between the core diagnostic genes and atopic dermatitis as well as allergic rhinitis **(A–E)**. Bubble plots showing the correlation between the expression levels of diagnostic hub genes and immune cell infiltration scores in AR, based on the MCPcounter algorithm. Genes analyzed include CD274 **(A)**, CYP2E1 **(B)**, FOLH1 **(C)**, SERPINB4 **(D)**, and SPRR1B **(E)**. **(F–J)** Bubble plots showing the correlation between the expression levels of diagnostic hub genes and immune cell infiltration scores in AD, based on the MCPcounter algorithm. Genes analyzed include CD274 **(A)**, CYP2E1 **(B)**, FOLH1 **(C)**, SERPINB4 **(D)**, and SPRR1B **(E)**.

In the AR cohort, CD274, SPRR1B, CYP2E1, and SERPINB4 exhibited strong positive correlations with dendritic cells and natural killer cells, suggesting their potential involvement in antigen presentation and innate immune activation. In contrast, FOLH1 showed a significant negative correlation with neutrophils and B cells (Figures 8A–E). Notably, NK cell infiltration was markedly elevated in AR patients compared with controls, further supporting the role of these core genes in modulating innate immune responses in AR pathogenesis (Supplementary Figure S2A). In the AD cohort, CD274, SPRR1B, CYP2E1, and SERPINB4 were significantly positively correlated with T cells and dendritic cells, reinforcing their potential roles in adaptive immunity and antigen processing. In contrast, FOLH1 did not display notable associations with any immune cell types (Figures 8F,J). Consistent with these findings, dendritic cells were significantly elevated in AD patients relative to controls, implicating their involvement in antigen presentation and disease progression (Supplementary Figure 2B).

Collectively, these results highlight a shared immune infiltration signature between AR and AD, with core diagnostic genes reflecting key elements of immune dysregulation. These genes may thus serve not only as reliable diagnostic biomarkers but also as mechanistic indicators of immune activation across both allergic conditions.

### 3.9 Construction of the mRNA–miRNA regulatory network of the core diagnostic genes

**Figure 9:**
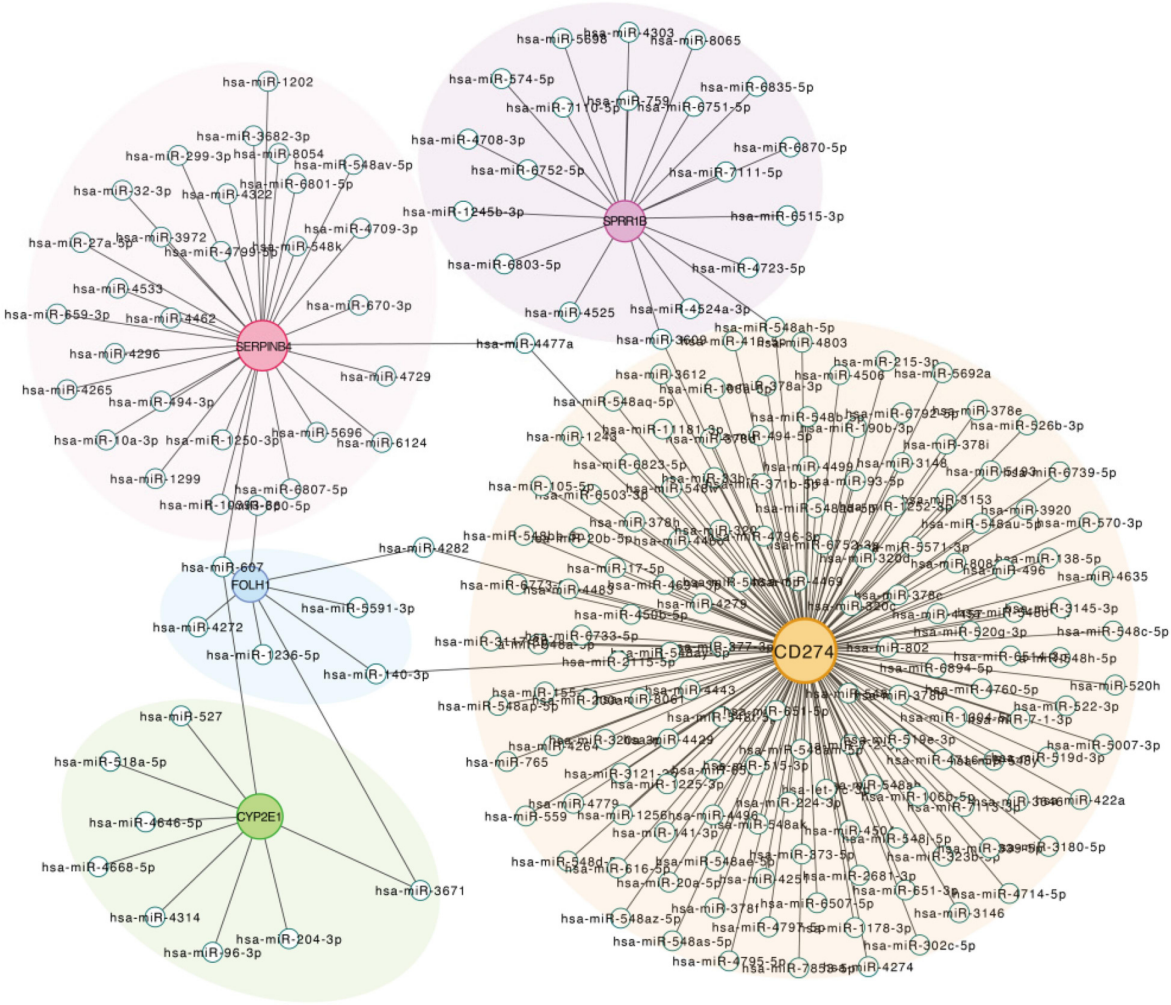
Construct the mRNA–miRNA regulatory network of the core diagnostic genes mRNA–miRNA regulatory network. The edge nodes represent different miRNAs, and the center nodes with gene names marked in different colors are displayed.

To explore the potential posttranscriptional regulatory mechanisms of the identified diagnostic genes, we constructed an mRNA–miRNA regulatory network. The network revealed that each hub gene is targeted by multiple miRNAs, suggesting complex layers of regulatory control. Notably, CD274 was associated with the highest number of predicted miRNA interactions, implying a central regulatory role. Other genes, such as SERPINB4, SPRR1B, and CYP2E1, were also regulated by distinct sets of miRNAs, further highlighting the gene-specific regulation landscape (Figure 9). These findings provide additional insight into the posttranscriptional modulation of diagnostic biomarkers in allergic diseases.

### 3.10 Drug–gene target network of the core diagnostic genes

**Figure 10:**
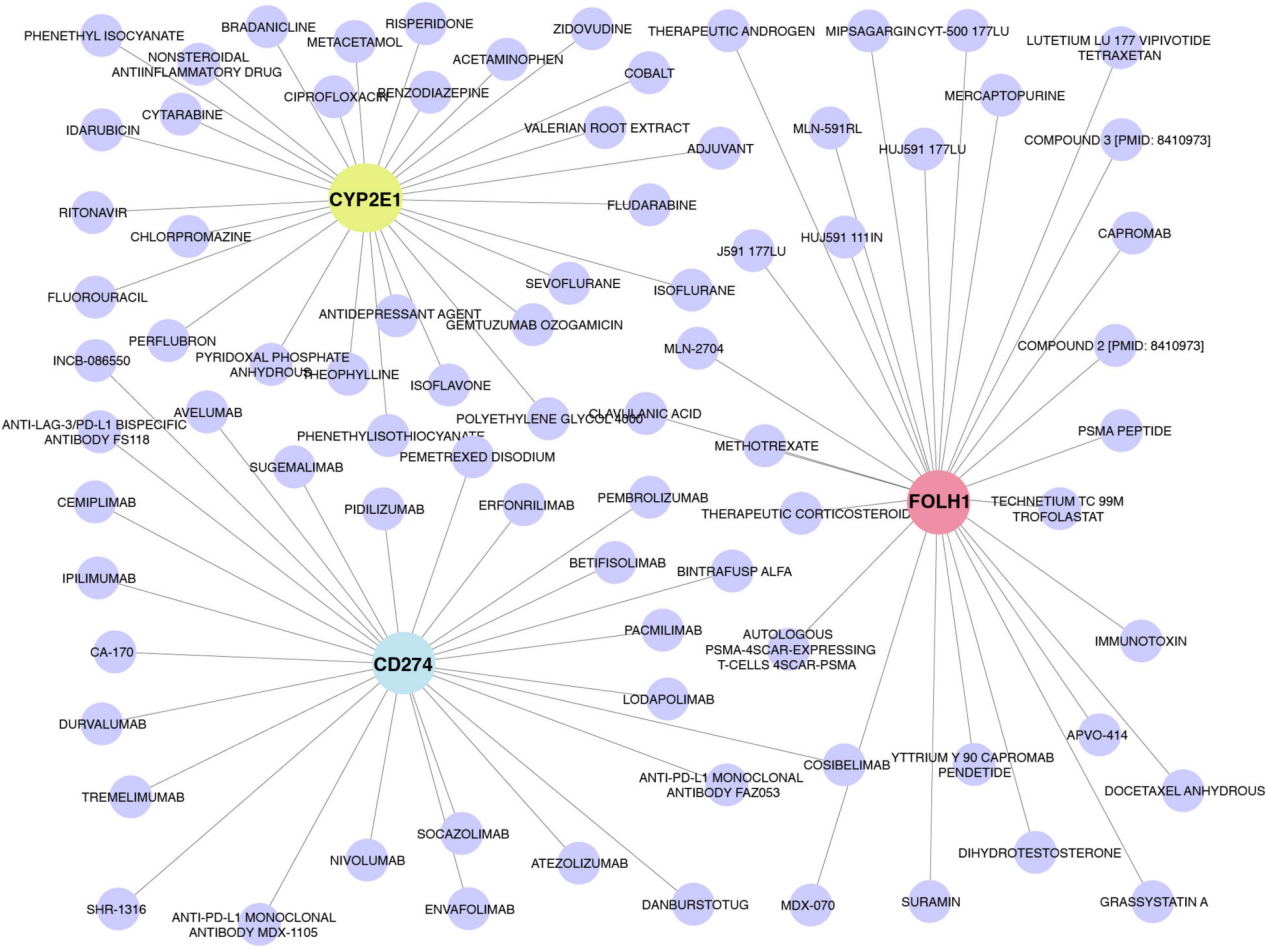
Drug–gene target network of the core diagnostic genes. The blue nodes represent different drugs, and the nodes with gene names marked in different colors are displayed.

To investigate the therapeutic relevance of the identified diagnostic biomarkers, we constructed a drug–gene interaction network using validated drug–target databases (Figure 10). The results revealed that several hub genes, particularly CD274, CYP2E1, and FOLH1, were associated with multiple approved or investigational drugs. These interactions suggest that the identified biomarkers not only hold diagnostic potential but may also serve as druggable targets for future therapeutic intervention in allergic diseases.

## 4 Discussion

The clinical co-occurrence of AR and AD has been widely reported in epidemiological studies, suggesting a close connection in their pathophysiological mechanisms (19, 20). However, despite the well-recognized clinical overlap, the molecular basis underlying this association remains insufficiently explored. In this study, we conducted an integrative bioinformatics analysis to investigate the shared biomarkers and potential mechanistic cross talk between AR and AD.

We first identified DEGs from transcriptomic datasets of AR and AD patients. A total of 36 overlapping DEGs were identified, suggesting common molecular disturbances between the two diseases. Subsequent PPI network construction revealed a subset of core interacting genes, and functional enrichment analysis indicated that these genes were involved in processes such as extracellular matrix organization, calcium ion signaling, and oxygen transport. These pathways are known to be implicated in barrier dysfunction and immune activation in allergic diseases (21).

Through machine learning analysis, five diagnostic hub genes (CD274, SERPINB4, CYP2E1, SPRR1B, and FOLH1) were identified as potential biomarkers for both AR and AD. ROC analysis demonstrated high diagnostic accuracy across validation cohorts, underscoring the reliability of these biomarkers in distinguishing allergic disease states. These genes were further confirmed to have consistent expression patterns across datasets, suggesting their potential utility in clinical biomarker panels.

Notably, immune infiltration analysis using the MCPcounter algorithm revealed that most hub genes were positively correlated with key immune cell populations, particularly dendritic cells, T cells, and natural killer cells. In the AR cohort, CD274, SPRR1B, and CYP2E1 showed strong positive correlations with dendritic cells and NK cells, implying a possible role in innate immune activation and antigen presentation (22). In the AD cohort, stronger associations were observed between hub genes and T cells, aligning with the chronic T-cell-mediated nature of AD. These results support a shared immunological mechanism across AR and AD, where both innate and adaptive immune cells are recruited and activated during disease progression.

Among the hub genes, SERPINB4 and SPRR1B have been reported to be involved in epidermal stress responses and skin inflammation (23–26). CD274 encodes PD-L1, an immune checkpoint molecule that may regulate local inflammation through modulation of T-cell activity (27). CYP2E1 is involved in oxidative metabolism and may contribute to the oxidative stress observed in chronic inflammation, while FOLH1 encodes an enzyme involved in folate metabolism, with possible immunomodulatory effects (28, 29). These genes represent diverse biological functions, including epithelial barrier integrity, immune regulation, and metabolic processes, all of which are known contributors to allergic inflammation.

Further pathway enrichment analysis confirmed the involvement of immune-related pathways, including Th1/Th2 cell differentiation, cytokine–cytokine receptor interaction, and IL-17 signaling, all of which are established contributors to allergic disease pathogenesis (10). These findings provide mechanistic evidence supporting the immunological and transcriptomic overlap between AR and AD.

Increasing evidence indicates that miRNAs shape allergic inflammation by modulating Th2 cytokine signaling and epithelial barrier integrity. For example, miR-19a and miR-155 are upregulated across allergic conditions and enhance IL-4/IL-13 production by repressing negative regulators (30–32). Consistent with this biology, our mRNA–miRNA network shows that each hub gene is targeted by multiple miRNAs, implicating miRNA dysregulation in the shared transcriptional program of AR and AD. Notably, our drug–gene network includes agents already used in routine care, such as corticosteroids (intranasal for AR; topical/systemic for AD) and methotrexate (systemic for refractory AD), lending face validity to the network and underscoring its translational relevance (33–35). Our analysis similarly showed that CD274, CYP2E1, and FOLH1 interact with multiple approved or investigational drugs. These observations suggest that the five-gene signature is not only an adjunct molecular marker but also points to therapeutic targets that could ameliorate both AD and AR.

In summary, this study identified key shared molecular markers between AR and AD through transcriptomic integration and machine learning analysis. The five core diagnostic genes demonstrated strong diagnostic value and were significantly associated with immune cell infiltration in both diseases. Our further exploration of the gene regulatory network and prediction of drug targets has provided valuable insights for the development of therapeutic approaches targeting relevant targets. These results not only enhance our understanding of the molecular basis of AR and AD comorbidity but also provide a foundation for the development of shared diagnostic tools and potential therapeutic targets.

Future studies should validate these findings in larger cohorts and at the protein level, including independent AD-only and AR-only cohorts to assess disease-specific relevance. In addition, future validation should explicitly stratify participants by treatment status at the time of sampling to quantify potential therapy-related perturbations. Moreover, functional experiments are needed to clarify the roles of the hub genes. In addition, single-cell omics of AD and AR will help resolve the microenvironment and cell-type-specific expression.

Overall, our results contribute to a growing body of evidence that supports the existence of shared pathophysiological mechanisms across allergic diseases and offer new directions for early diagnosis and intervention.

## 5 Conclusion

In this study, from the perspective of comorbidity, we employed integrative bioinformatics and machine learning to explore shared mechanisms between AR and AD. We identified five core diagnostic hub genes (CD274, SERPINB4, CYP2E1, SPRR1B, and FOLH1) as shared biomarkers, with high diagnostic accuracy for both diseases. These genes are linked to immune cell infiltration and key allergic pathways, supporting their potential as shared diagnostic tools and therapeutic targets.

## Data Availability

The original contributions presented in the study are included in the article/Supplementary Material; further inquiries can be directed to the corresponding author.
