# Supplementary material for: Shared diagnostic genes and potential mechanism between allergic rhinitis and atopic dermatitis revealed by integrated transcriptomic analysis and machine learning
Source: Front Allergy. 2025 Nov 21;6:1686370. doi: 10.3389/falgy.2025.1686370 (PMC12678292; doi:10.3389/falgy.2025.1686370)
Supplement: Supplementary file 1 [file Datasheet1.pdf]

Supplementary Material

1 Supplementary Figures and Tables

1.1 Supplementary Figures 1

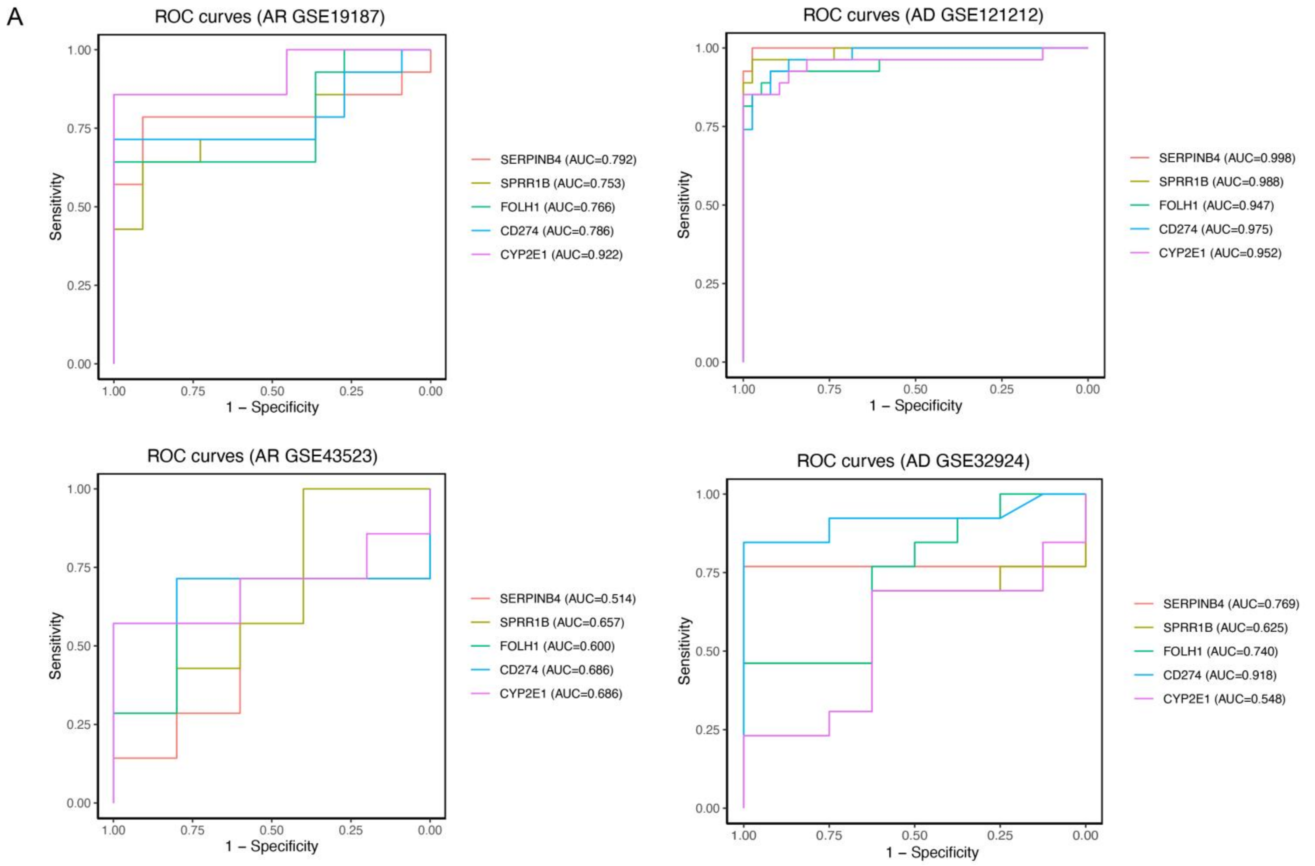

Supplementary Figure 1. ROC curves of core diagnostic genes. (A). ROC curves of core diagnostic genes in the corresponding cohort.

1.2 Supplementary Figures 2

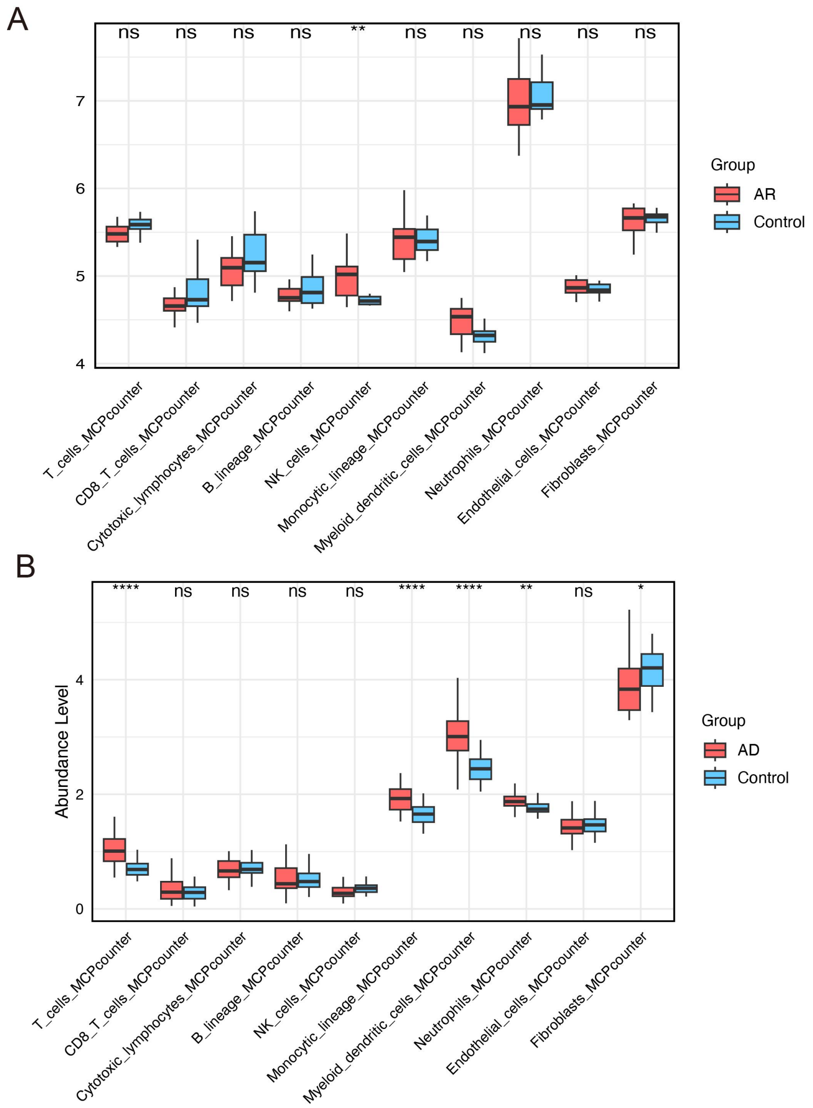

Supplementary Figure 2. Immune infiltration analysis of AR and AD. (A). Boxplot of immune cell score in AR and control groups. (B). Boxplot of immune cell score in AD and control groups. Statistical significance was assessed using a t-test (* P < 0.05, ** P < 0.01, *** P < 0.001).

1.3 Supplementary Table 1

Dataset	Disease Type	Disease Sample(n)	Control Sample(n)	Annotation
GSE121212	Atopic Dermatitis	27	38	Lesional skin of patients with atopic dermatitis or healthy controls
GSE32924	Atopic Dermatitis	13	8	Lesional skin of patients with atopic dermatitis or healthy controls
GSE19187	Allergic Rhinitis	14	11	Patients with isolated rhinitis or healthy controls
GSE43523	Allergic Rhinitis	7	5	Patients with seasonal allergic rhinitis or healthy controls
In house clinical samples	Atopic Dermatitis&Allergic Rhinitis	5	5	Patients with both allergic rhinitis& atopic dermatitis or healthy controls (Age: 30-42 years / male:female ratio of 1:1)

Supplementary Table 1. Demographic information table

1.4 Supplementary Table 2

Dataset	OOB estimate of error rate
GSE121212	4.62%
GSE32924	16%

Supplementary Table 2. OOB estimate of error rate of RF
